# The future of research software is the future of research

**DOI:** 10.1016/j.patter.2025.101322

**Published:** 2025-07-11

**Authors:** Neil P. Chue Hong, Selina Aragon, Simon Hettrick, Caroline Jay

**Affiliations:** 1 EPCC, University of Edinburgh, Edinburgh, UK; 2 School of Electronics and Computer Science, University of Southampton, Southampton, UK; 3 School of Engineering, University of Manchester, Manchester, UK

## Abstract

The use of software is near-ubiquitous in research, yet it is still underrecognized despite changes in policy and practice. Notwithstanding many successful initiatives to improve the culture around research software, the authors argue that it is essential that the development of research software anticipates changes in the research landscape and continues to support the many different people who use it.

## Main text

### Introduction

In 2010, the Software Sustainability Institute (SSI) was founded in the United Kingdom with the goal of supporting and improving the software used in research. At that time, software was already prevalent throughout academic research, although its importance was often poorly recognized. Software fulfills many different roles^1^: the instrument generating data or simulating a model; the tool managing, analyzing, or visualizing the data; the platform integrating components; the infrastructure enabling computation; or the facilitator assisting research collaboration. There have even been attempts to define “research software,” which “includes source code files, algorithms, scripts, computational workflows, and executables that were created during the research process or for a research purpose.”^2^ Now more than ever, it is important to ensure that research software is used and developed responsibly, ethically, and collaboratively to ensure that everyone can benefit from the outcomes of research.

The SSI believes that helping individuals and institutions understand the vital role that software plays in research will accelerate progress in all fields and academic endeavors. Over the years, the SSI has advocated for, and developed an understanding of, the fundamental importance of research software, its developers and users, its requirements, and how software advances research in the UK and across the world.^3^^,^^4^ It has done this by recognizing that people—and people working together—are the key to scaling up the ability to produce more reliable, reproducible, and reusable software and, ultimately, that better software leads to better research.

This has led to initiatives supported by the SSI becoming part of the rich fabric of research, some of which are described here. Each has come out of necessity but also demonstrates a commitment to embedding itself within the community so it can thrive and grow independent of the SSI’s direct support.

### Recognition and career paths for research software engineers

Research relies on software, but historically, there has been no formal role recognizing this. It was during a discussion of this systemic shortcoming at an SSI Collaborations Workshop in 2012 that the term “research software engineer” (RSE) was coined. Since then, the SSI has sought to establish the role of RSEs in research through building community; raising awareness among universities, research institutions, and policymakers; and campaigning for a recognized RSE career path.

In 2013, the SSI held the first workshop for RSEs, attended by 56 self-described RSEs. In 2016, the SSI founded the RSE Network to support the creation of new RSE groups, which have increased in number from a single group at University College London to over 40 groups across the UK and nine other national RSE associations across the world. In 2019, a new professional body was established: the Society of Research Software Engineering.^5^ With this final step, the SSI succeeded in creating a wholly independent, community-led organization that underpins the RSE community and safeguards its future, with over 700 paying members at the time of writing.

There are now over 6,700 RSEs worldwide participating in the RSE community on Slack, helping increase the uptake of good coding practices, and improving the effectiveness and quality of research. But there remains a shortage, due in part to the lack of a clear training path. To address this, the SSI is working with the RSE community to develop open training materials covering topics that emphasize the unique aspects of working in research teams, which will allow more candidates from a wider range of backgrounds to embark on highly valuable and rewarding RSE careers.

### Access to software training for all

Too few researchers use software effectively, leading to inefficient research that lacks reproducibility and reusability. In seeking to remedy this issue, starting in 2012, the SSI chose to partner with The Carpentries rather than develop a rival set of training materials. This embraces the SSI’s “collaborate, don’t compete” ethos and has led to a fruitful partnership that has revolutionized access to research software training within the UK and around the world.

The initial Software Carpentry workshops aimed to teach researchers practices that were “good enough” and easy to apply. Over time, competence has grown, and more complex training options have been offered. To support this evolution, the SSI helped to establish a network of over 300 qualified instructors in the UK who could deliver this openly licensed material—now, many researchers and RSEs can pass on these skills to others. The SSI has also empowered those engaged in research to write and deliver their own training material by codeveloping a Collaborative Lesson Development course^6^ that teaches pedagogical principles and provides a reusable format so materials can be shared easily with other instructors within the community.

There is also a need for a wide diversity of training resources to ensure that everyone can identify and develop the skills they need to work with research software. Over the years, the SSI has helped launch and support initiatives including The Turing Way handbook for reproducible, ethical, and collaborative data science (facilitated by SSI Fellows Malvika Sharan, Sarah Gibson, Becky Arnold, and many others)^7^; CarpentriesOffline training in areas where there is limited or no Internet access (SSI Fellows Jannetta Steyn and Colin Sauze); and Research Software Camps in Spanish, Italian, and Ukrainian to address the English-centric nature of much training in this area.

### Making software visible

The SSI has been integral to the development of international research software standards, bringing together colleagues from across the wider research community to share ideas, obtain feedback, and encourage the adoption of policies. The development of principles for software citation, for example, had substantial roots in events organized and run by the SSI, starting with a green paper published by the SSI in 2012 entitled “How to cite and describe software”^8^ based on experiences working with different projects. Work by SSI Fellows Robin Wilson, Rob Haines, and Stephan Druskat across several workshops and hackathons led to development of the Citation File Format (CFF).^9^ Support for software citation using CFF was announced in 2021 by GitHub, Zenodo, and Zotero, and 10 years after their inception, guidance^10^ and metadata standards^11^ based on these principles were adopted by major publishers.

More recently, similar international community efforts have led to the extension of the FAIR Principles for data to research software,^12^^,^^13^ bringing together more than 500 collaborators from 110 organizations and 34 countries. In the UK, the Hidden REF began as a one-off competition organized by the SSI in 2020 with the goal of celebrating all research outputs and everyone who is involved in their creation. It has since become a widely recognized campaign drawing on support from professional societies and publishers and building a passionate and highly engaged grassroots community, which contains many people who are not typically included in the development of research policy.

This continued advocacy and support of initiatives addressing key challenges to software visibility has resulted in the implementation and adoption of new policies across the research infrastructure, ensuring that the people who create software can be properly recognized.

### The future of research software is the future of research

Shifts in the wider research landscape, driven by open research and the reproducibility crisis, have enshrined software and code in research policy. At the same time, use of software has shifted from being mostly confined to computational sciences to being involved in all areas of research. But more recently, the pace of change in research practice has accelerated, driven by major changes in government policy, an understanding of the impact of research on the climate emergency, and the rapid innovation of artificial intelligence and machine learning. This necessitates new approaches to support research software and the research infrastructure (physical, human, and digital) that enables it.

Traditionally, digital and physical research infrastructure have been considered separately—a viewpoint that no longer works when software is used to manage and control research laboratories, vehicles, and equipment. Infrastructure, by its very nature, evolves slowly, but research practice moves quickly. Good infrastructure accelerates research, but right now, it isn’t clear whether researchers and institutions are taking this on board. Consider, for instance, the issues that labs might have faced in 2010 if network ports were in the wrong places. Now, research infrastructure will need to be driven by data engineering, metadata, and the ability to integrate software and data.

At the individual level, the emergence of AI coding assistants has revolutionized the way that people develop code. Now that research software engineering has been built up as a profession, and RSE groups have risen as a way for researchers and RSEs to work together to develop code, tools are available that enable any researcher to explore a problem computationally. The need for an RSE to develop code on behalf of a researcher is disappearing, but there is still a requirement for a skilled professional to provide the assurance that the software is correct.

Generative AI is the accelerant for a much wider change in research, heralding a new era where experimental design will radically change. Reducing the time to discovery has benefits but cannot be done at the loss of transparency and explainability. Equally, it is impossible to talk about increased access to computing without highlighting its environmental impact. The SSI is addressing this through support for the Green DiSC certification scheme (led by SSI Fellow Loïc Lannelongue), which provides a road map for research groups and institutions who want to tackle the environmental impacts of their computing activities, and the Green RSE Special Interest Group (SIG), which brings RSEs and researchers who code together to understand how to reduce the environmental impact of research software. After all, well-engineered code reduces the amount of computing required.^14^

### Working together is the only effective way to do research

In 2023, at the annual Research Software Engineering Conference that forms one of the lasting outcomes of the SSI’s work, the Research Software Development Principles^15^ were published. These nine principles drew on the SSI’s experience, and the input of its collaborators, to recognize that the sustainable development of research software is multifaceted and therefore cannot be tackled by addressing a single goal or approach, or by a single project or organization, recasting research software development as being “societally good.” From helping your team to helping your peers to helping the world, the principles provide a short set of goals that every research software project should strive for, which—if projects and organizations collectively adopted them—would make research software better for all.

So much of what the SSI does is grounded in working together. The team is drawn from many different places, disciplines, and backgrounds. Its fellows and collaborators come from across the world, contributing to a vibrant ecosystem united by a common goal: advancing the sustainability of software in research and beyond. This has resulted in lasting culture change and a resilient community of passionate people. At the most recent Collaborations Workshop, where once again a diverse group of people came together to tackle shared challenges, it was no surprise that topics included improvements in carbon literacy; safeguarding of research and cultural data; mental health first aid; the importance of diversity, equity, and inclusion; and, of course, the future of research software.

It is hard to predict where the world will be in 2040, but it is clear that it will benefit from empowering everyone engaged in research to fully harness the potential of software to enable excellent research.

## Acknowledgments

This work was initially funded by the Engineering and Physical Sciences Research Council (EP/H043160/1) and subsequently funded by all seven UK research councils (EP/N006410/1 and EP/S021779/1). The Software Sustainability Institute is currently funded by UK Research and Innovation through their Digital Research Infrastructure program (AH/Z000114/1). The authors thank all past and present staff, fellows, and collaborators for their valued contributions—this work would not have been possible without them.

## Author contributions

Conceptualization, N.P.C.H., S.A., S.H., and C.J.; writing – original draft, N.P.C.H, S.H., and C.J.; writing – review & editing, N.P.C.H.; funding acquisition, N.P.C.H., S.A., S.H., and C.J.

## Declaration of interests

S.H. is a member of the *Patterns* advisory board.
